# Imaging Transformer for MRI Denoising: a Scalable Model Architecture that enables *SNR* ≪ 1 Imaging

**Published:** 2025-04-13

**Authors:** Hui Xue, Sarah M. Hooper, Rhodri H. Davies, Thomas A. Treibel, Iain Pierce, John Stairs, Joseph Naegele, Charlotte Manisty, James C. Moon, Adrienne E. Campbell-Washburn, Peter Kellman, Michael S. Hansen

**Affiliations:** 1. Microsoft Research, Health Futures, Redmond, WA, USA; 2. National Heart, Lung and Blood Institute, National Institutes of Health, Bethesda, MD, USA; 3. Institute of Cardiovascular Science, University College London, London, UK; 4. Barts Heart Centre, Barts Health NHS Trust, London, UK

## Abstract

**Purpose:**

To propose a flexible and scalable imaging transformer (IT) architecture with three attention modules for multi-dimensional imaging data and apply it to MRI denoising with very low input SNR.

**Methods:**

Three independent attention modules were developed: spatial local, spatial global, and frame attentions. They capture long-range signal correlation and bring back the locality of information in images. An attention-cell-block design processes 5D tensors ([B, C, F, H, W]) for 2D, 2D+T, and 3D image data. A High Resolution (HRNet) backbone was built to hold IT blocks. Training dataset consists of 206,677 cine series and test datasets had 7,267 series. Ten input SNR levels from 0.05 to 8.0 were tested. IT models were compared to seven convolutional and transformer baselines. To test scalability, four IT models 27m to 218m parameters were trained. Two senior cardiologists reviewed IT model outputs from which the EF was measured and compared against the ground-truth.

**Results:**

IT models significantly outperformed other models over the tested SNR levels. The performance gap was most prominent at low SNR levels. The IT-218m model had the highest SSIM and PSNR, restoring good image quality and anatomical details even at SNR 0.2. Two experts agreed at this SNR or above, the IT model output gave the same clinical interpretation as the ground-truth. The model produced images that had accurate EF measurements compared to ground-truth values.

**Conclusions:**

Imaging transformer model offers strong performance, scalability, and versatility for MR denoising. It recovers image quality suitable for confident clinical reading and accurate EF measurement, even at very low input SNR of 0.2.

## Summary statement

We present a novel imaging transformer that offers superior performance, scalability, and versatility for MRI denoising. It can recover high-quality images suitable for confident clinical reading, even at very low input SNR levels of 0.2.

## Introduction

MRI denoising aims to recover signals from low signal-to-noise ratio (SNR) acquisitions. Ideally, MRI should provide high SNR, resolution, and contrast to visualize anatomical structures for disease identification. However, imaging physics imposes trade-offs—SNR decreases with smaller pixel size and shorter acquisition times, such as in undersampled parallel imaging.

Deep neural networks have emerged as powerful denoisers (1–12), enabling faster acquisitions with improved diagnostic accuracy (4,9,13). Most published work exploited variants of convolutional neural networks (CNN) (5,6,8,10–12,14). Examples include Resnet (15) to denoise the diffusion weighted brain scans, feed-forward CNNs to reduce noise in neuro T1, T2 and fluid attenuated 3D MRI (11), and U-net to enhance knee MRI (10). These CNN solutions significantly improved over traditional filtering-based methods (2).

Recent advances in computer vision favor transformer-based architectures (4,16,17), which outperform CNNs in classification, detection, and segmentation tasks by capturing long-range dependencies and adapting to input variations (2,18–22). The reason can be attributed to a few fundamental differences between the architectures. First, the attention coefficients in transformers are computed on-the-fly for every input sample, potentially enabling them to adapt better, compared to convolutional kernels’ fixed parameters after training. Second, transformers are better at capturing long-range dependencies between signals and enable larger effective receptive fields, experimentally consistent with improved performance (23).

Originally developed for language modeling (24), transformers face challenges in imaging due to the quadratic complexity of attention mechanisms, making full-image attention computationally expensive. Vision Transformer (ViT) (25) introduced image tokenization, splitting the whole volume into patches and computing attention over tokens rather than pixels, surpassing CNN baselines (26). However, its full-attention design is computationally expensive and lacks inductive bias for locality, which is crucial for imaging where neighboring pixels are highly correlated. The Shifted Window Transformer (Swin) (21) addressed this by computing attention within local windows overlapped with each other by shifting, reintroducing some inductive bias. Originally designed for classification and segmentation, Swin has been adapted for denoising (22), proving effective for 3D arterial spin labeling MRI, outperforming ViT and ResNet baselines (4).

Both ViT and Swin simply stacked their transformer modules into a feed-forward backbone. As the denoising task requires pixel-wise prediction on the original image resolution, this design does not take advantage of multi-resolution scheme to balance computation and model capacity (27). Other studies proposed to replace convolution modules in a U-net architecture by transformer modules (19), to outperform feed-forward backbone.

A good imaging architecture enables global pixel interactions to capture long-range correlations while benefiting from locality and reducing computational complexity. Versatility should be provided to handle diverse data formats, such as 2D, 2D+T or 3D (there are limited sets of 3D+T scenarios which are not targeted here). We propose a new set of transformer modules that are collectively termed *Imaging Transformer (IT)*, applied to MRI denoising. It was designed to decouple intra-frame local, global, and inter-frame correlations. These modules process images flexibly, taking a 5D tensor [B, C, F, H, W] as input and output. Here, B is batch size, C is channels, H and W are height and width, and F represents time or depth or slice in 3D volumes. For 2D imaging, F is unitary.

**Figure 1.**
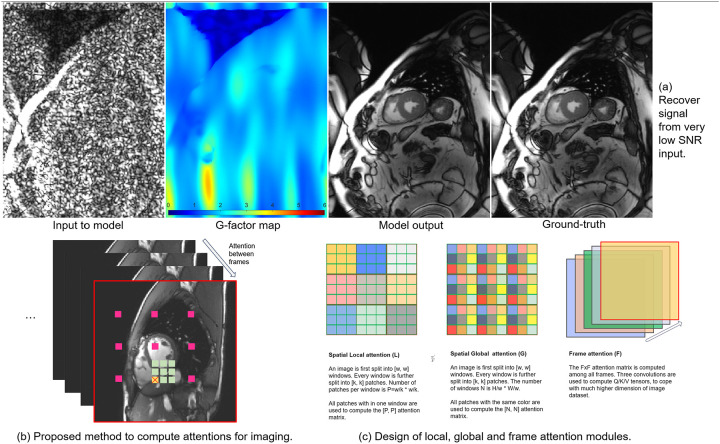

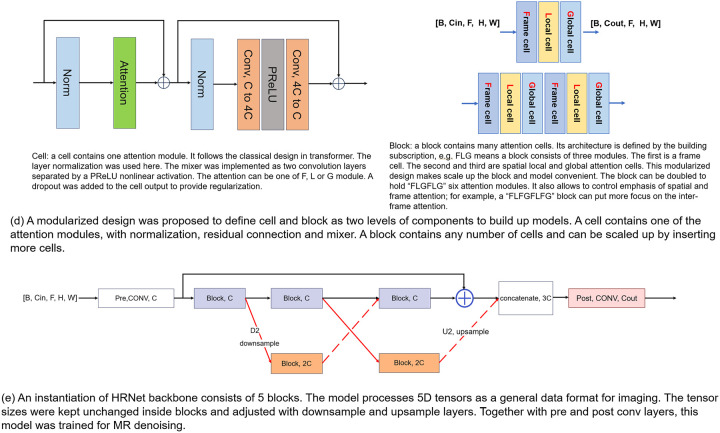
Imaging attention modules and model design. (a) Proposed model recovered signal faithfully from very low SNR input. (b) We proposed the 5D tensor [B, C, F, H, W] as a key data format to represent imaging data, for 2D, 2D+T or 3D acquisition. Full attention across three dimensions can incur very high computing costs and may not be optimal for not exploiting the locality of information. Instead, intra- and inter-frame attention can be separately computed. For the spatial (along H, W) attention, a target patch (marked in yellow) can take in information from both close neighbors (marked in green) and remote patches (marked in pink). We showed this approximation method mitigates quadratic complexity of full attention, brings back inductive bias and is very effective in MR denoising. (c) Three attention modules are illustrated. Spatial attention splits an image to multiple windows. Each window is further divided into patches. Local attention computes the attention matrix with all patches in one window. Global attention computes the attention over the corresponding patches from all windows. For example, one attention is computed over all blue patches and another attention is computed over all red ones. Inter-frame correlation is captured with frame attention. (d) Following the classical transformer design, a cell holds an attention module, layer normalization and the mixer, with the skip connections. A cell can hold either G, L or T attention, to aggregate information from different regions. A block is a container for many cells. Model can be scaled up in size by inserting more blocks or inserting more cells into every block. (e) A HRNet backbone was instantiated to hold five blocks at two resolution levels. The number of channels after downsample was doubled. Every block takes in a 5D tensor and produces another 5D tensor as the output. By doing so, all components can be concatenated. For the denoising task, we also add pre- and post-convolution to convert input 3 channels (real, imag and g-factor) to C=64 internal channels. Output conv will produce 2 channels for complex data.

Our model effectively recovers signals from extremely low SNR inputs (Figure 1a). By decomposing full attention into local (L), global (G), and frame (F) modules, we reduce computational complexity to linear scaling with the size of data volume. Each module operates independently, allowing customization for capacity and flexibility by inserting additional IT blocks. This design provides more explicit control to where the model should pay more attention. For example, more frame modules can be inserted into the block to enhance inter-frame modelling. In this study, we trained IT-based denoising networks ranging from 27M to 218M parameters on a high-resolution network (HRNet (28), Figure 1d) backbone using 206,677 2D+T cardiac cine series and tested on 7,267. The IT models were compared with other baselines. Model outputs were evaluated by two expert cardiologists for diagnostic confidence. The EF from model images were compared to the ground-truth measurements.

## Materials and Methods

### Data collection

Data for this retrospective study was from the NIH Cardiac MRI Raw Data Repository, hosted by the Intramural Research Program of the National Heart, Lung and Blood Institute. All data were curated with the required ethical and/or secondary audit use approvals and guidelines that permitted retrospective analysis of anonymized data without requiring written informed consent for secondary usage for the purpose of technical development, protocol optimization, and quality control. All data were fully anonymized and used in training without exclusion.

The training data consists of breath-held retro-gated cardiac cine imaging from 3T clinical scanners (MAGNETOM Prisma, Siemens AG Healthcare). A dataset of 206,677 cine series (6,160,700 frames) from 16,220 patients was compiled. Imaging used a balanced steady-state free precession (B-SSFP) sequence with typical parameters as the following: field-of-view 360×270 mm^2^, matrix 256×144, bandwidth 977 Hz/pixel, flip angle 50°, parallel acceleration R=2, echo spacing 2.97ms, echo time 1.28ms, and 30 phases over 7–10 heartbeats. Scans included two-chamber (CH2), three-chamber (CH3), four-chamber (CH4), and short-axis stack (SAX) views. Raw k-space signals were stored for reconstruction. The test set contained 7,267 series from 525 patients, with no overlap between training and test data.

### Imaging Transformer (IT) modules

IT modules process a 5D tensor and output another 5D tensor by computing attentions along spatial and frame dimensions. Given the input tensor x, the Query (Q), Key (K), and Value (V) tensors are first computed. To introduce locality bias and support variable matrix sizes, three convolutions are used to compute Q, K, and V. These convolution kernels are learnable parameters, differing from standard attention that uses linear layers. This design allows freely adjusting the number of channels through the network. As shown later, the channel is proportional to dimensions of q/k/v tensors which controls the capacity of imaging attention. That is, models can be scaled up by increasing number of channels as well as adding more attention modules.

#### Spatial local attention (L)

With the Q/K/V tensors computed, they are divided into attention windows containing multiple patches. Given the window size [w,w] and patch size [p,p], the number of patches per window is $P=\frac{w}{p}\cdot \frac{w}{p}$. The name “spatial local” implies the attention computation is limited to all patches within one attention window on every [H,W] 2D frame. Every patch has $p^{2}$ pixels. Flattening all pixels in a patch across the C channel gives the pixel vector $v\in R^{Cp^{2}\times 1}$. Stacking all P vectors as a data matrix D gives:

$$D_{\text{local}}=\left[\begin{array}{c} v_{0}^{T}\\ v_{1}^{T}\\ \vdots \\ v_{P-1}^{T} \end{array}\right]\in R^{P\times Cp^{2}}$$

These above-mentioned steps were repeated for Q,K and V, resulting in $D_{Q},\;D_{K}$ and $D_{V}$. A larger C increases attention capacity which allows more information to be stored in K and V. The attention matrix is computed between $D_{Q}$ and $D_{K}:A\left(D_{Q},D_{K}\right)=\text{softmax}\left(D_{Q}D_{K}^{T}/\sqrt{Cp^{2}}+B\right).\;A$ is the $R^{P\times P}$ attention coefficient matrix. B is the relative positional bias matrix (29). The output for this window is $A\cdot D_{V}$. This attention computation is performed for every window with the batched matrix computation. The final output is reshaped back to be a 5D tensor. A multi-head attention version is achieved by splitting v for every head and computing attention matrixes for all heads.

#### Spatial global attention (G)

Global attention captures dependencies between remote regions, while the local attention focuses on neighboring patches. As shown in Figure 1c, global attention assembles the data matrix over corresponding patches from all attention windows. Given the window size [w,w], the number of attention windows is $N=\frac{H}{w}\cdot \frac{W}{w}$. Stacking patches gives the global attention data matrix D:

$$D_{\text{global}}=\left[\begin{array}{c} v_{0}^{T}\\ v_{1}^{T}\\ \vdots \\ v_{N-1}^{T} \end{array}\right]\in R^{N\times Cp^{2}}$$

The attention computation is over the same color patches from N windows (Figure 1c), resulting the $R^{N\times N}$ attention coefficient matrix which is applied to the *value* tensor.

We also experimented with random shuffling the patches in a window, then computed attention. But this extra step had no impact on model performance.

#### Frame attention (F)

Correlations between frames in imaging are strong. Processing every 2D frame independently is not optimal. The number of frames in a scan is in general much less than the number of pixels. It is feasible to perform full attention computation along the frame dimension. Given the 5D tensor with M frame, the data matrix is assembled for all frames:

$$D_{T}=\left[\begin{array}{c} v_{0}^{T}\\ v_{1}^{T}\\ \vdots \\ v_{M-1}^{T} \end{array}\right]\in R^{M\times CHW}$$

$v_{i}\in R^{\text{CHW}\times 1}$ contains all pixels in the i-th 2D frame flattened. The resulting attention coefficient A is a M×M matrix. All output frames are computed by multiplying A to the value tensor.

### Backbone and training

As shown in Figure 1d, following the classical design (24), we integrate attention modules with other layers into a Cell which consists of an attention module, layer normalizations (30), skip connections (15), and a mixer. The attention module can be G, L, or F. The mixer includes layer normalization, PReLU activation (31), and two convolution layers: the first scales the channels by a factor of 4, and the second restores the original channel count. Multiple cells form a processing Block, and we use acronyms G, L, or F to indicate the attention module type in the block. For example, a FLG block consists of frame, local, and global attention cells. The model can be scaled by adding cells; for instance, a FLGFLG block has six cells, doubled the size. A dropout (32) of 0.1 is applied to cell outputs for regularization. All convolution kernels are 3×3 with padding of 1.

Attention modules, cells, and blocks form the three levels of building blocks for the full imaging transformer model. We use the HRNet backbone, having more processing blocks on the highest resolution representations by avoiding early-stage downsampling (Figure 1e). For imaging where fine-grained details are crucial, this architecture favors signal recovery with high fidelity. We replace the convolution layers of the original HRNet CNN with imaging transformer blocks.

The SNRAware training method (33) synthesizes training samples from high-SNR data, adding MR imaging noise augmented with g-factor maps of varying accelerations. It normalizes the noise level to unity through SNRUnit (34) reconstruction instead of normalizing signal levels (35). Inputs include complex image series and g-factor maps, and outputs are denoised complex images. The input channel $C_{in}$ is 3 (real and complex values with the g-factor map), and the output channel $C_{\text{out}}$ is 2. The encoding channel C is 64. The training set uses 95% for training and 5% for validation. Training setup is as follows: the Sophia optimizer (36), a one-cycle learning rate scheduler (37), peak learning rate 1e-5, betas 0.9 and 0.999, epsilon 1e-8, 160 epochs, Pytorch 2.6 (38), a cluster of 128 AMD MI300X GPUs, each with 192GB RAM. Further details are in Appendix E1 (supplement).

### Evaluation

**Figure 2.**
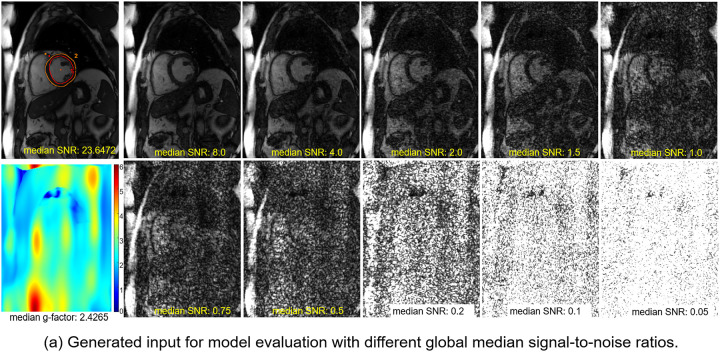

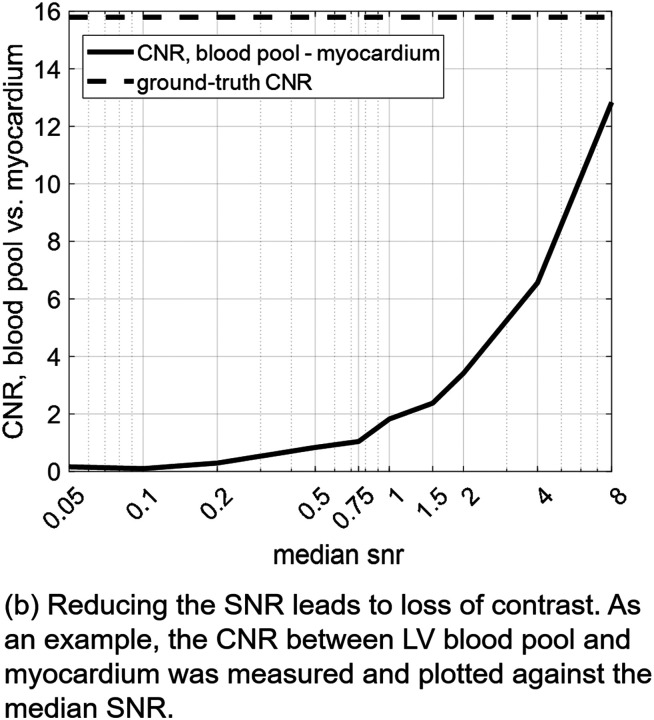
A test data with N=10 levels of signal-noise-ratio. (a) The GT data has a high SNR of 23.6. By sampling noises and amplifying them with g-factor maps, a set of 10 input series with SNR from 8.0 to 0.05 was created test the model. At low SNR end, e.g. SNR<1, the anatomical features are lost to the elevated noise and blood-myocardial contrast decreases. (b) The blood-myocardial contrast-to-noise ratio was plotted against the input SNR. The elevated noise reduces the CNR asymptotically towards zero and makes the image uninterpretable.

A large test dataset was created with 7,267 series from 525 subjects, including CH4, CH2, and SAX stacks for each. All test data were retro-gated cine scans from 3T scanners. The median signal-to-noise ratio (SNR) was used to evaluate image quality, with raw SNR measured using the SNRUnit reconstruction, which normalizes noise standard deviation (34). R=4 g-factor maps $g_{R=4}$ were computed for each test case (33). The global median SNR is computed as $\text{median}\left(SNR_{\text{original}}/\sqrt{1.0+{\left(nn\cdot g_{R=4}\right)}^{2}}\right)$. Here nn is the added noise standard deviation. Ten target global SNR levels were tested: [0.05, 0.1, 0.2, 0.5, 0.75, 1.0, 1.5, 2.0, 4.0, 8.0], and SD of added noise was computed for each level. As shown in Figure 2, at low SNR, myocardium and blood-pool contrast were lost, and anatomical structures became uninterpretable to human readers (also see Supplement Movie 1).

Four imaging transformer models were trained by scaling up the HRNet backbone: a) IT-27m with block FLG (27 million parameters); b) IT-55m with block FLGFLG; c) IT-109m with block FLGFLGFLGFLG; d) IT-218m with block FLGFLGFLGFLGFLGFLGFLGFLG. Seven baseline models were trained for comparison: ViT3D (25,26) for 27m and 55m, Swin3D (22) for 55m, CNNT (39) for 27m and 55m and convolutional HRNet (28) for 23m and 45m. Their parameters were chosen to match the two smaller IT models. Peak SNR (PSNR, $10\cdot {log}_{10}\left(\frac{MAX^{2}}{MSE}\right)$) and structural similarity index measure (SSIM) (40) were computed for model outputs. MSE is the mean square difference and MAX is the maximal image value, set to 2048.0.

Two cardiologists (RHD and TAT, >10 years’ experience, level III EACVI) reviewed model outputs for 55 randomly selected test cases. GT and model outputs at ten SNR levels were presented together as movies (examples in Supplement Movie 2). Each expert independently picked the lowest SNR offering sufficiently good quality for clinical interpretation. Good quality was defined as no difference in clinical interpretation compared to GT, with adequate contrast, preservation of important anatomical features, and the same subjective assessment of cardiac function.

Cardiac function was quantitatively evaluated by applying a pre-trained, clinically validated cine segmentation model (41), to the GT and model outputs at all SNR levels. This model segmented the left ventricle and myocardium. The model EF was compared to GT values.

The segmentation masks from the GT were used to measure blood-pool and myocardial signal levels, comparing them to the model outputs to assess how well the model recovered the blood-myocardial contrast.

## Results

**Figure 3.**
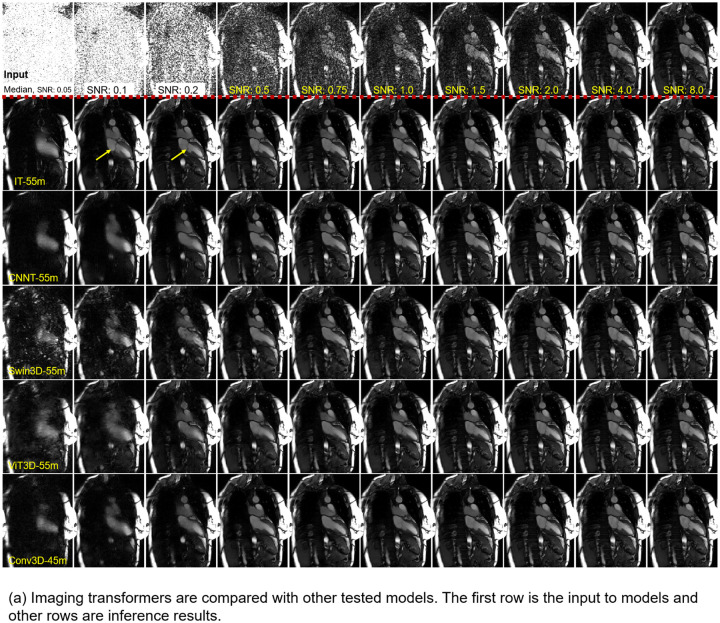

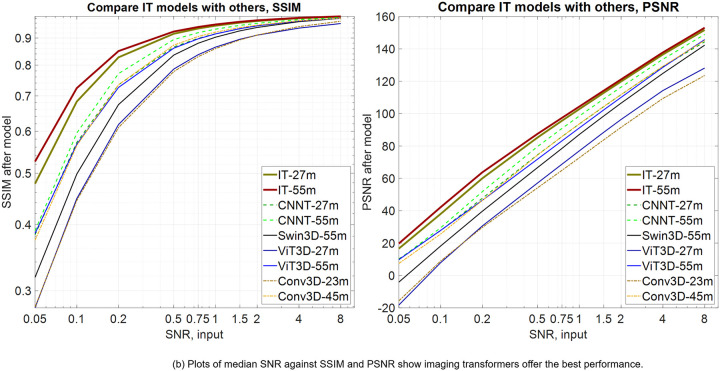
Comparison to other models (also in Supplement Movie 3). (a) The IT-55m model was compared to other tested models with matching number of parameters. The top row is the input from low SNR of 0.05 to high 8.0. All models worked well at higher SNR end, but imaging transformers showed noticeable lead in overall image quality, especially the recovery of anatomical details when SNR is 0.2 or lower. At SNR 0.05, outputs of all models are non-diagnostic, showing the limit of model and space for further improvement. (b) The test set was processed with all models. SSIM and PSNR were computed over all SNR levels and plotted. The IT-55m model outperformed others consistently across the wide SNR range. The performance gap was more substantial at the low SNR.

Figure 3 presents model comparison results. IT-55m was evaluated against other models with similar sizes for SSIM and PSNR at various SNR levels. Both SSIM and PSNR decreased with lower SNR, with IT models outperforming others across all levels. At higher SNR (8.0), all models exhibited excellent SSIM (>0.95), but noticeable differences appeared at lower SNR. For instance, at SNR 0.2, IT-55m had the highest SSIM of 0.8504, outperforming Swin3D-55m (0.6739) and ViT3D-55m (0.7261). Smaller models performed worse; IT-27m had SSIM 0.8276, and ViT3D-27m had 0.6179. Figure 3a shows an example test case for all models and SNR levels. At higher SNR, all models produced good quality and well delineated anatomical details (e.g. the mitral valve). At lower SNR, IT-55m outperformed others, consistent with SSIM and PSNR metrics. Supplement Movie 3 provides zoomed-in videos.

**Figure 4.**
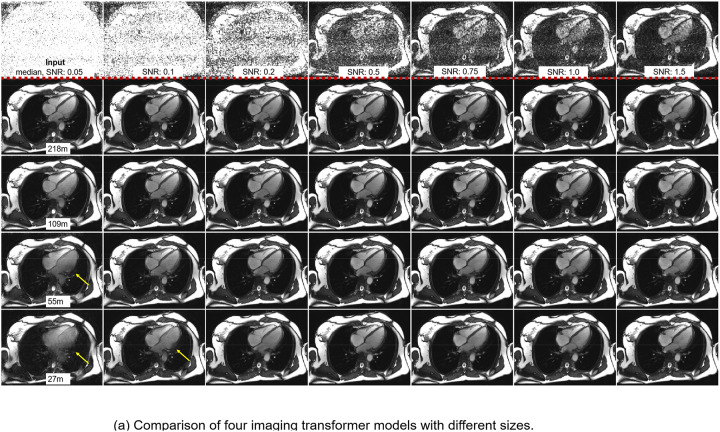

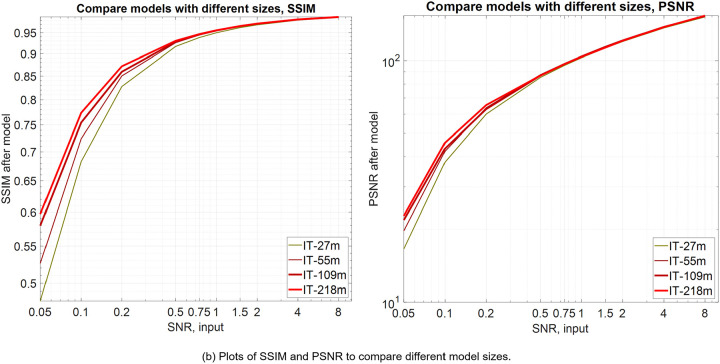
Comparison of four imaging transformer models of different sizes (also in Supplement Movie 4). (a) Four IT models were applied to a test example. The bigger model had the best image quality at the low SNR. (b) The curves show that scaling up the model size improves the performance.

Figure 4 compares the four IT models, showing that performance improved with model size, particularly at low SNR. At SNR 0.2, IT-218m achieved an SSIM of 0.8717, higher than IT-109m (0.8596), IT-55m (0.8504), and IT-27m (0.8276). At SNR 0.1, the SSIM values were 0.7736, 0.7544, 0.7240, and 0.6829, respectively. Figure 4a provides an example for the four models, and Supplement Movie 4 shows corresponding videos. Larger models better preserved anatomical details and tissue contrast. The IT-218m showed loss of details at SNR 0.05, indicating its limit.

Supplement Movies 5, 6, and 7 show IT-218m performance across different cardiac views at various SNR levels (2.0, 0.5, 0.2, 0.1). The model was robust across the SNR range, with significant restoration of image quality, even for fine details like valve motion and papillary muscles, even at low SNR.

Two cardiologists reviewed outputs from 55 test cases, identifying the lowest SNR level for confident clinical interpretation. The median among both experts was that the IT model restored image quality for diagnosis at SNR 0.2 and above. Supplement Movie 8 demonstrates noisy inputs, model outputs, and ground truths at SNR 0.2.

**Figure 5.**
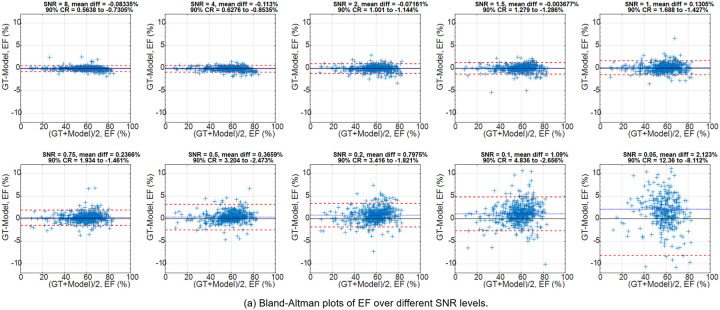

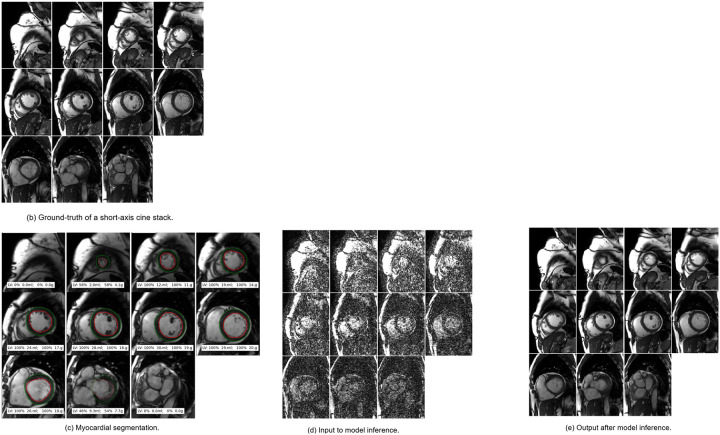
The ejection fraction measured on IT-218m outputs were compared to ground-truth values. (a) The Bland-Altman plots of EF, GT vs. model over all SNR levels, show that model measurement is correct. The 90% CR increased with lower SNR levels. (b-e) An example of EF measurement is shown here. The cine analysis model was applied to the short-axis stacks of the GT and model outputs, to segment the myocardium and blood pool. The image quality was greatly improved after model, allowing the cine analysis to work well on the model outputs.

The cine analysis model was used to segment the LV and myocardium, and compute EF. Figure 5a shows Bland-Altman plots of EF for IT-218m and ground truth at all SNR levels. The mean deviation at SNR 8.0 was −0.08335%, and at SNR 0.2, increased to 0.7975%. The 90% confidence range (CR) increased with lower SNR. For context, the cine analysis model has a test-retest reproducibility of 4.6% for EF (41), which was higher or on par with the CR up to SNR 0.2. Figure 5b–e gives an example to illustrate the analysis. Supplement Movie 9 shares the corresponding movies.

Smaller models like IT-27m had inferior image quality and higher errors in EF estimates. Figure E1 [supplement] shows the EF Bland-Altman plots for IT-27m. At SNR 8.0, IT-27m showed a mean deviation of −0.09215%, comparable to IT-218m. At SNR 0.2, the error increased to 4.463%, much higher than IT-218m. The same trend was found for CR. When SNR was lower than 0.5, the IT-27m had higher scatter of errors than the IT-218m. Figure E1c [supplement] compared the 90% CRs of IT-27m against the IT-218m.

**Figure 6.**
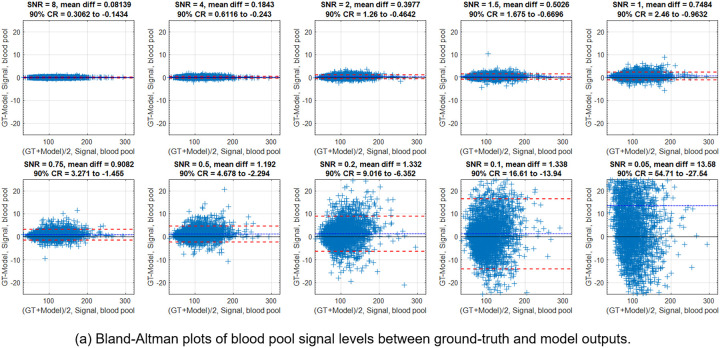

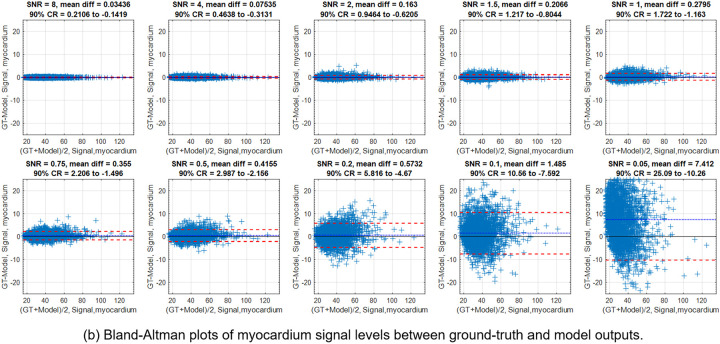

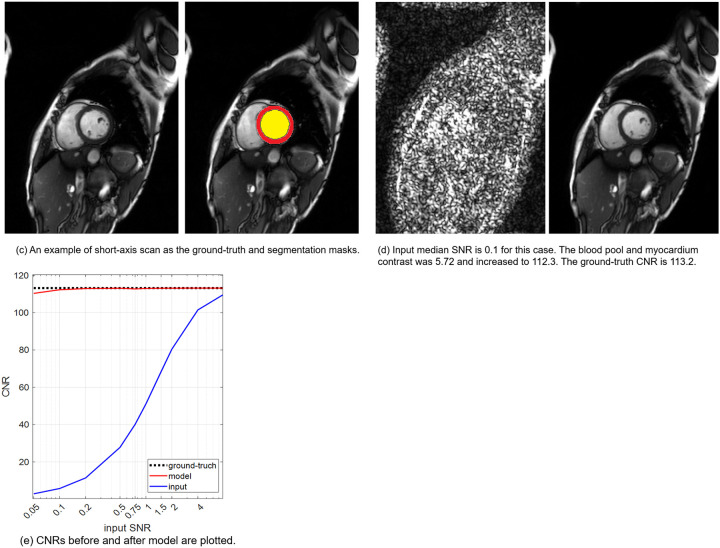
The blood pool and myocardial signal levels were measured with segmentation masks generated on the GT data. (a-b) The Bland-Altman plots for the blood signal and the myocardium. (c) An example of segmentation mask overlaid on the image. (d) For the starting SNR 0.1, the CNR was reduced from 113.2 to 5.72. After model, the CNR level was restored to be 112.3. (e) The CNR was measured for all SNR levels. The model was able to restore the contrast until ~SNR 0.2. With even lower SNR, the resulting CNR started to deviate from the ground-truth, but was still much higher than starting values.

Figure 6a and 6b give the Bland-Altman plots of blood and myocardial model signals against the GT. The mean signals for blood and myocardium were 105.7 and 37.0. The deviation in blood signal was less than 0.08% at SNR 8.0 and 1.26% at SNR 0.2. For myocardium, they were 0.09% and 1.55%. As a result, the blood-myocardial contrast was restored, as shown in Figure 6e and Supplement Movie 10.

The model’s robustness against varying acceleration factors is demonstrated in Figure E2 (supplement) with g-factor maps from R=2 to R=5. At R=5, the g-factor noise amplification was severe. The model effectively denoised spatially amplified noises across a range of starting SNRs, with performance starting to slip only at SNR ~0.2. Supplement Movie 11 and 12 show videos before and after model application.

## Discussion

Recovering signals from noisy data is crucial for high-performance imaging. A key finding is that large IT models can recover image quality sufficient for clinical reading, even at very low SNRs. When noise is five times higher than the signal, the blood-myocardial contrast is lost, and fine anatomical structures are noninterpretable. However, the model still recovers signal faithfully, producing EF estimates, showcasing the power of large IT models.

The core innovation of imaging transformers lies in decoupling full attention into spatial local, spatial global, and frame modules. This reduces quadratic complexity, offers versatility and flexibility, and enables focused attention on intra- and inter-frame dimensions. A module–cell–block architecture allows for model scaling. Using a HRNet backbone, IT models were trained on a large MR denoising dataset, outperforming baselines using ViT, Swin, and CNNs, with the performance gap widening at lower SNRs. Scaling IT models from 27 million to 218 million parameters improved performance across all SNR levels.

Other studies in language modelling have attempted to address quadratic complexity of the classical scale-dot-product attention (42) through sparse (43–45) or low-rank approximations (20,46,47). The IO-aware Flash-attention (48,49) speeds up attention without approximation using kernel fusion, re-computation, and multi-threading. For images, MaxViT (20) decouples full attention of a 2D image into local and remote components, which is the same spatial splitting as our approach. However, we use convolutions to maintain locality bias, handle varying matrix sizes, and reduce number of parameters, offering an effective low-rank approximation by limiting the attention matrix computation.

With a large training dataset, we showed that IT models can scale for better performance. Larger models achieved higher SSIM and PSNR across SNR levels, and IT-218m produced more accurate EF estimates. The error for the 27m model increased 5.6× at SNR 0.2, highlighting the importance of scaling for accurate biomarker estimation. Unlike other studies focused on SSIM or loss metrics, our work specifically evaluated clinical relevance. To our best knowledge, no other studies have verified biomarker estimation can benefit from model scaling. One other study scaled the U-net for MR reconstruction (50) to measure SSIM. The language model scaling was assessed for cardiac MR tasks (49) to compare the cross-entropy loss.

This study has limitations. We focused on introducing the IT model and evaluating performance across a range of SNR levels with available ground-truth data and did not target more generalization cases. While we assessed EF here, other biomarkers are also relevant. Clinical applicability could be tested in more imaging sequences and conditions. Lastly, although we demonstrated scaling up to 218 million parameters, further scaling requires advancements in model software and training methods. More computing is needed for larger datasets. All of these are topics for future research.

## Supplementary Material

1

## Data Availability

The authors thank the Intramural Research Program of the National Heart, Lung, and Blood Institute for the data obtained from the NIH Open-Source Cardiac MRI Raw Data Repository.

## Key points:

1. We introduced a novel and flexible imaging transformer architecture for MRI denoising. The key innovation lies in decomposing full attention across imaging data into intra-frame local, global, and inter-frame attention computations.
2. The imaging transformer model recovered image quality sufficient for confident clinical readings and accurate measurement of ejection fraction (EF) even for inputs with an SNR of 0.2, where images were uninterpretable without denoising.
3. Effects of scalability were experimentally verified with performance improved as model size increases. The larger model had more accurate EF measurement than smaller ones. Training was performed on a large dataset of 206,677 cine series and testing was on 7,267 samples. The imaging transformer outperformed all seven convolutional and transformer baselines.

## Abbreviations

MRI: magnetic resonance imaging
SNR: signal-to-noise ratio
CNN: convolutional neural network
ViT: vision transformer
Swin: shifted window transformer
IT: Imaging Transformer
HRNet: high-resolution network
EF: ejection fraction
GT: ground-truth
B-SSFP: balanced steady-state free precession
LV: left ventricular
CH2: two-chamber
CH3: three-chamber
CH4: four-chamber
SAX: short-axis stack
SD: standard deviation
PSNR: peak signal-noise-ratio
SSIM: structural similarity index measure
CNR: contrast-to-noise ratio
CR: confidence range
