# Supplementary material for: Imaging Transformer for MRI Denoising: a Scalable Model Architecture that enables SNR ≪ 1 Imaging
Source: ArXiv. 2025 Apr 13:arXiv:2504.10534v1. Preprint. [Version 1] (PMC12306840)
Supplement: 1 [file NIHPP2504.10534V1-supplement-1.pdf]

Supplemental Appendices

Appendix E1. Information for deep learning models

A key design choice is the 5D tensor [B, C, F, H, W]. Because all cells consume and produce the 5D tensor, any number of cells can be inserted into a block. A block also respecting this convention, can be linked together, to compose model backbone.

Different backbone designs are available. Choices include a stack of blocks or simple feed-forward network or adapting the successful CNN backbones and converting them into a transformer model. In this study, we picked the high-resolution network as the backbone. This design maintains a long process branch with the original matrix size, which is suitable for denoising tasks, because the pixel-level prediction is required. In contrast, if the aggregated prediction, such as segmentation or classification, was the target, maintaining original matrix size may not be necessary. In those cases, U-net type backbone with early downsampling can reduce computing cost.

Downsampling was implemented with patch merging (21) followed by a convolution to alter the number of channels if needed. The upsampling was implemented with a linear interpolation followed by a convolution.

Recent study showed the small patch size led to improved performance (51). The minimal patch is a single pixel. It can lead to an attention sequence length of a few thousand. In this study, we chose patch size 2×2 and window size 8×8. For Swin3D and ViT3D, a window includes the third dimension. The 3D window size there was 8×8×16 for H, W, and F.

Supplemental Figures

Figure E1. Comparison of EF measurements for small and large models.

The improved image quality from scaling up the model sizes was propagated to the more accurate EF measurements. The Bland-Altman plots for IT-27m were given there. Both the mean error and the 90% CR were much higher than IT-218m model. At SNR 0.2, the EF error was 4.463% for IT-27m, and 0.7975% for IT-218m. The bar plot showed the confidence range where clear gaps were seen at low SNRs.

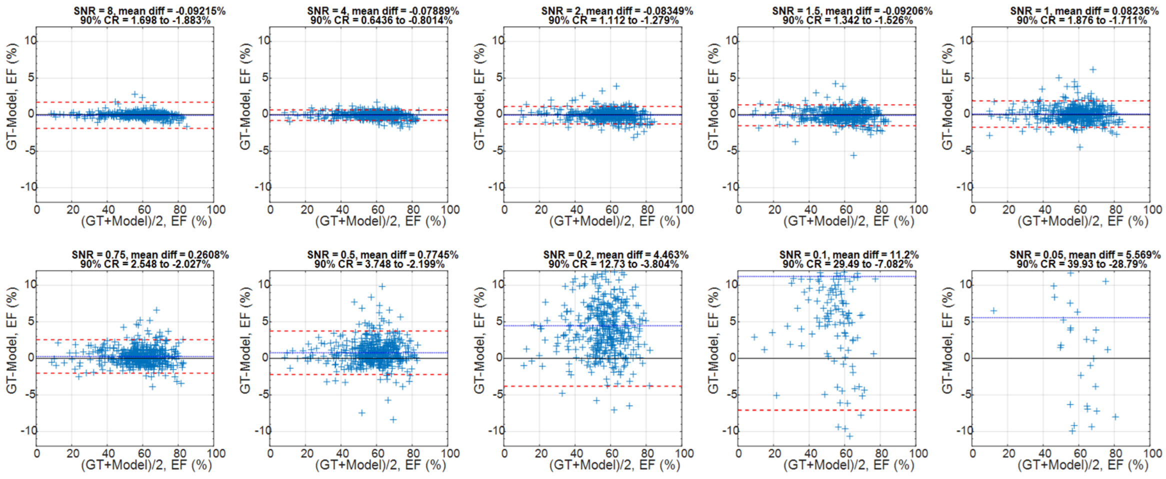

(a) Bland-Altman plots of input SNR vs. EF for **IT-27m**.

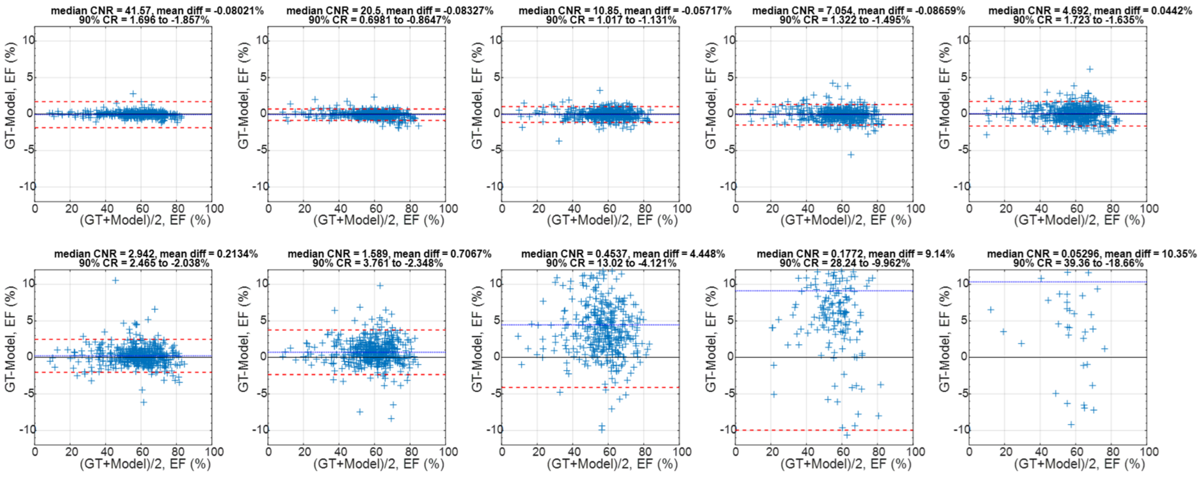

(b) Bland-Altman plots of input CNR vs. EF for **IT-27m**.

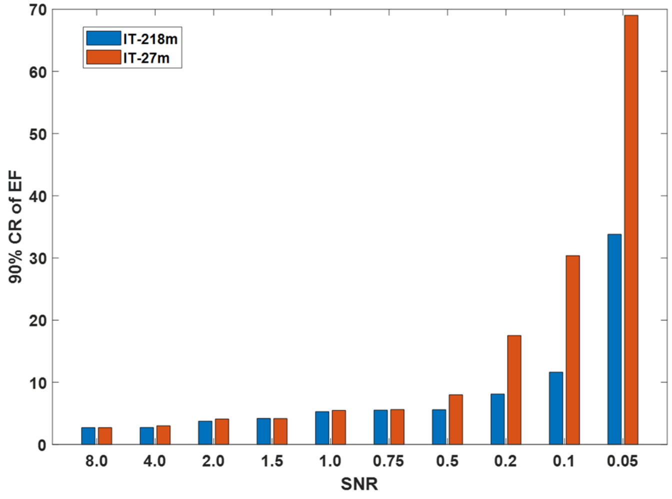

(c) Comparison of 90% CR of IT-218m and IT-27 models for EF.

Figure E2. Model performance on input data with spatially variant noise amplification from R=2-5 acceleration.

The model was robust to remove the spatial variant noise due to g-factor amplification. (a) A CH2 cine was corrupted for (b) g-factor maps from R=2 to 5 to create input data with median SNR from ~0.02 to 7.8. (c) Input data to the model at low SNR showed severely degraded image quality and loss of contrast. (d) The corresponding model outputs revealed the limitations of signal recovery. The output quality degrades visibly when input SNR was lower than ~0.2.

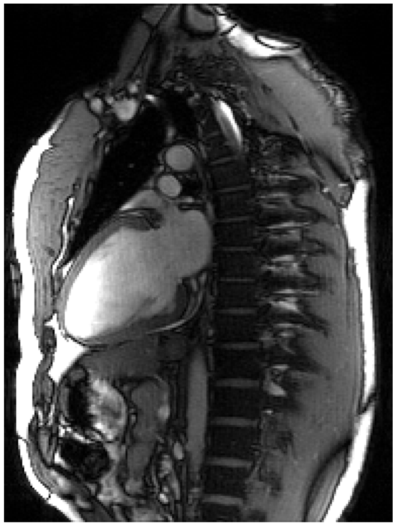

(a) Ground-truth CH2 cine.

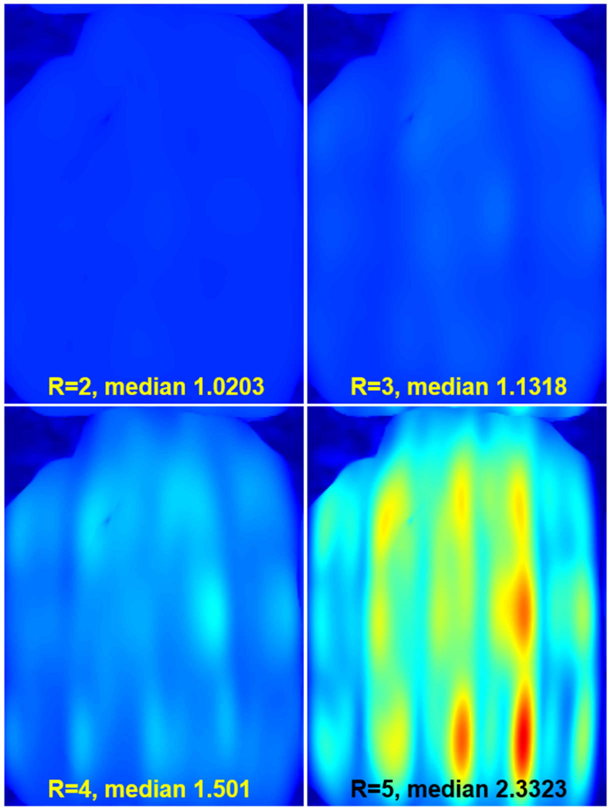

(b) G-factor maps for R=2 to 5, used for generating noise with spatial amplification.

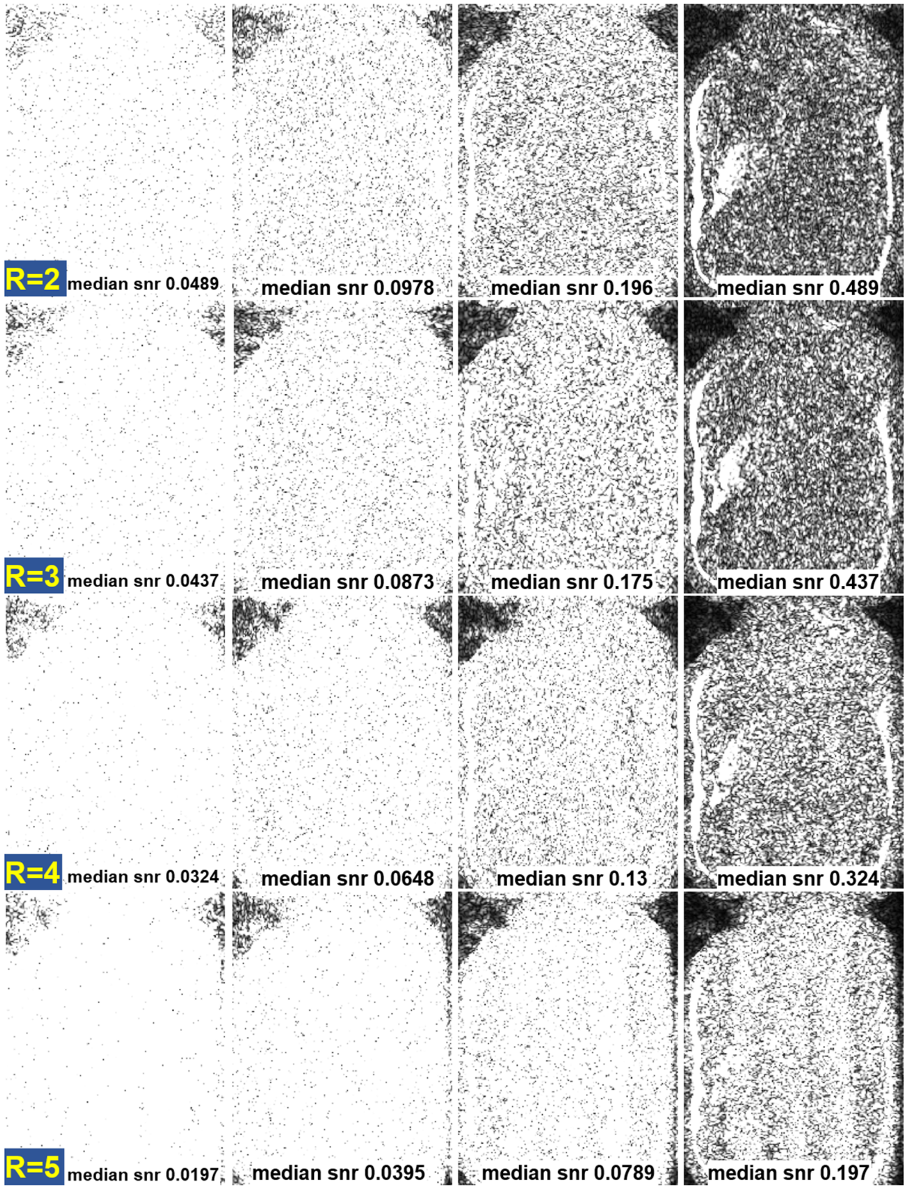

(c) Low SNR inputs for model inference.

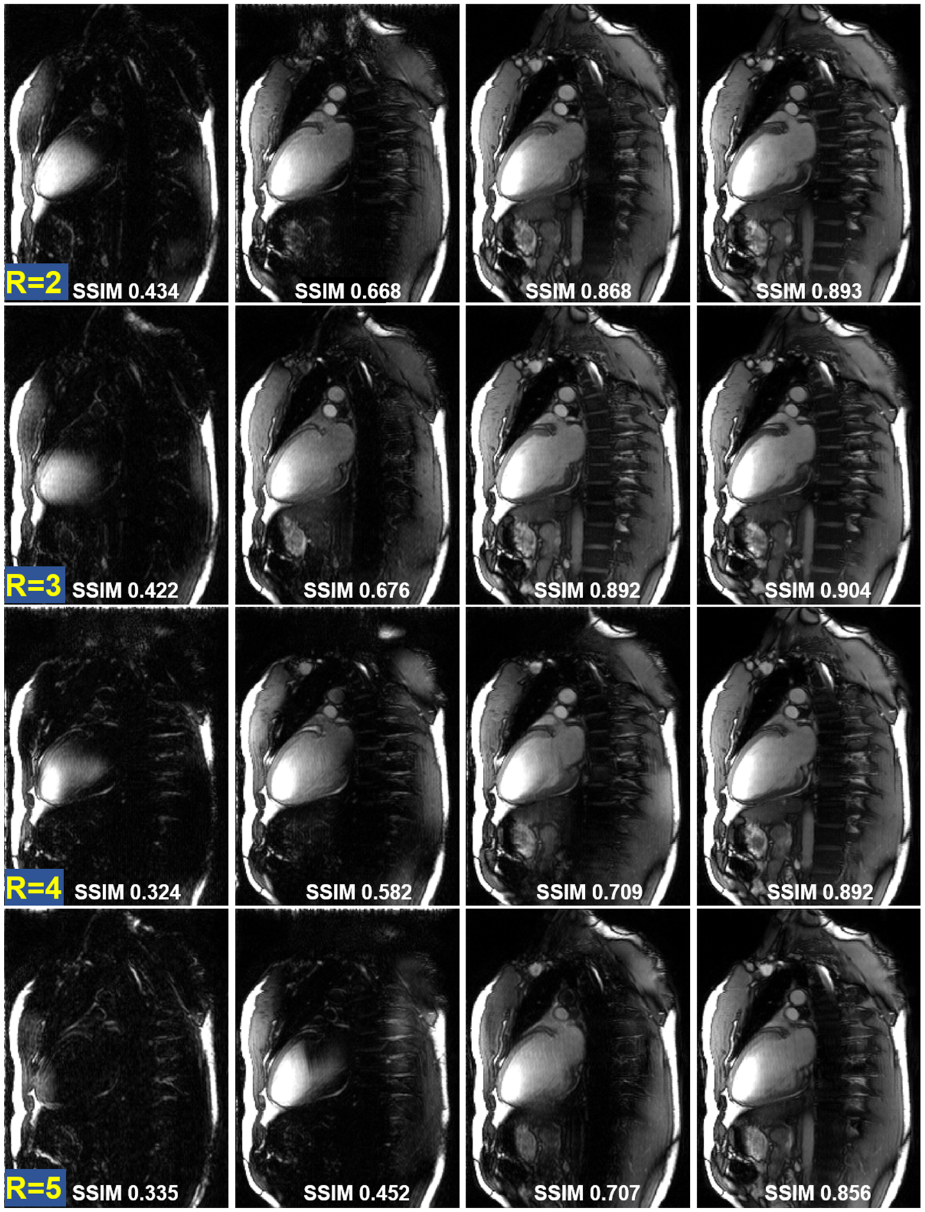

(d) Model outputs. SSIM was computed against the GT.

Supplemental Data

Movie 1: The movies correspond to the example in Figure 2.

Movie 2: Examples of ground-truths and model outputs, reviewed by cardiologists. All 11 movies were presented to the reviewers all together. The first one was the ground-truth and others were from the model.

Movie 3: The movies correspond to Figure 3. The right panel is the zoomed-in view around the beating heart.

Movie 4: The movies correspond to Figure 4. The lower panel is the zoomed version.

Movie 5: A four-chamber example of IT-218m model.

Movie 6: A short-axis example of IT-218m model.

Movie 7: A two-chamber example of IT-218m model.

Movie 8: More examples with input SNR level 0.2. The ground-truth is given on the right for reference.

Movie 9: The movies correspond to Figure 5. An example of short-axis stack processed by the model for the EF measurements. The pre-trained cine analysis model was applied to both GT and model output SAX stacks. Two EF estimates were computed, independently. If a model faithfully recovered image quality, its EF should agree with the ground-truth EF.

Movie 10: The movies correspond to Figure 6 for the blood and myocardial signal level measurement.

Movie 11: The movies correspond to Figure E2 (supplement), panel c. A set of examples were created for acceleration R=2 to 5 for SNR from 0.0197 to 7.82. The spatial variant noise was visible at R=4 or 5.

Movie 12: The movies correspond to Figure E2 (supplement), panel d. The model processed low SNR data and restored image quality over a wide range of input SNR and spatially variant noise amplification. For input SNR ~0.2 (two examples in red box), the SSIM of model were ~0.85. Further lower SNR led to decay of output quality, showing the current limitations of model.
